# Rethinking ME/CFS Diagnostic Reference Intervals via Machine Learning, and the Utility of Activin B for Defining Symptom Severity

**DOI:** 10.3390/diagnostics9030079

**Published:** 2019-07-19

**Authors:** Brett A. Lidbury, Badia Kita, Alice M. Richardson, Donald P. Lewis, Edwina Privitera, Susan Hayward, David de Kretser, Mark Hedger

**Affiliations:** 1 National Centre for Epidemiology and Population Health, RSPH, College of Health and Medicine, The Australian National University, Canberra, ACT 2601, Australia; 2 Paranta Biosciences Limited, Suite 549, 1 Queens Rd, Melbourne, VIC 3004, Australia; 3 CFS Discovery, Donvale Specialist Medical Centre, Donvale, VIC 3111, Australia; 4 Centre for Reproductive Health, Hudson Institute of Medical Research, Clayton, VIC 3168, Australia; 5 Department of Anatomy and Developmental Biology, School of Biomedical Sciences, Monash University, Clayton, VIC 3800, Australia

**Keywords:** myalgic encephalomyelitis, chronic fatigue syndrome, activin, pathology, biomarker, cytokine, machine learning, reference intervals

## Abstract

Biomarker discovery applied to myalgic encephalomyelitis/chronic fatigue syndrome (ME/CFS), a disabling disease of inconclusive aetiology, has identified several cytokines to potentially fulfil a role as a quantitative blood/serum marker for laboratory diagnosis, with activin B a recent addition. We explored further the potential of serum activin B as a ME/CFS biomarker, alone and in combination with a range of routine test results obtained from pathology laboratories. Previous pilot study results showed that activin B was significantly elevated for the ME/CFS participants compared to healthy (control) participants. All the participants were recruited via CFS Discovery and assessed via the Canadian/International Consensus Criteria. A significant difference for serum activin B was also detected for ME/CFS and control cohorts recruited for this study, but median levels were significantly lower for the ME/CFS cohort. Random Forest (RF) modelling identified five routine pathology blood test markers that collectively predicted ME/CFS at ≥62% when compared via weighted standing time (WST) severity classes. A closer analysis revealed that the inclusion of activin B to the panel of pathology markers improved the prediction of mild to moderate ME/CFS cases. Applying correct WST class prediction from RFA modelling, new reference intervals were calculated for activin B and associated pathology markers, where 24-h urinary creatinine clearance, serum urea and serum activin B showed the best potential as diagnostic markers. While the serum activin B results remained statistically significant for the new participant cohorts, activin B was found to also have utility in enhancing the prediction of symptom severity, as represented by WST class.

## 1. Introduction

The quest for a quantitative diagnostic and a specific marker for ME/CFS has yet to identify a reliable candidate, whether through routine pathology markers, or research efforts in immunology, microbiology, neuroscience and elsewhere. A number of cytokines, for example transforming growth factor-beta (TGF-β) and interleukin-10 (IL-10), have shown previous promise, but have not ultimately delivered a validated diagnostic test [1,2,3,4,5,6,7]. To the list of potential serum markers, we have recently added activin B, which was detected in a pilot research study involving volunteers recruited via CFS Discovery (Donvale Victoria) [8].

Activin B, along with activin A, is a member of the activin family of proteins, which belong to the TGF-β superfamily of growth and differentiation factors. Follistatin is a high-affinity binding protein for both activins, with diverse roles in physiology that include reproduction, haematopoiesis, immune cell development, as well as inflammation and immunity. The biology of activin A, at the time of writing, is better understood than that of activin B, although there is evidence of differences in relation to hepcidin regulation, associated receptor binding and SMAD signalling [9,10,11].

Following the activin findings from preliminary studies [8], this investigation aimed to validate these previous observations on a separate and larger population recruited by the same ME/CFS clinic in Melbourne. As well as the activin focus, other aims included applying the results from pathology and clinical testing, with and without activin B, to the pattern recognition algorithm random forest (RF), to identify wider marker patterns that separate ME/CFS cases from healthy controls. In addition to the development of activin B as a serum biomarker, a longer-term aim is to develop simpler diagnostic tools from routine data to assist health professionals diagnose ME/CFS.

The report herein examines the diagnostic potential of serum activin B, both individually and in combination with other blood, serum and urine markers considered for the assessment of research participants. The investigation directly compared the ME/CFS cases to healthy controls, but also examined the application of the weighted standing time (WST), as a measure of symptom severity, to stratify the ME/CFS cohort into mild to severe classes prior to analysis.

## 2. Materials and Methods

### 2.1. Participant Recruitment and Ethics Approval

The recruitment of research participants and associated procedures were described previously [8]. All the participants were recruited via CFS Discovery (Donvale, VIC, 3111), either via direct invitation to existing patients, or responses to advertising locally, and via social networking sites. Only participants with a previous ME/CFS diagnosis were recruited.

Human Ethics approval was granted by the ANU Human Research Ethics Committee (Approval No. 2015/193, approval date 29 June 2015), with approved consent forms and participant information provided to each potential participant. Inclusion in the study was allowed after signed consent was received by the researchers. Specific participant identifiers were not supplied to the researchers, and only known to the clinicians and clinic staff. Each research participant was given an identification code by the clinic, with age (at time of the appointment) and sex also provided. Eighty-five (85) participants were initially recruited for the ME/CFS cohort, with five eventually excluded due to comorbidities and/or difficulties attending the required appointments. Seventeen (17) healthy control (HC) participants were recruited too and underwent the same assessment as the ME/CFS cohort, giving a total study cohort size of 97 participants.

### 2.2. ME/CFS Assessment, Sample Collection and Tests

Each participant was examined by the CFS Discovery clinicians using the International Consensus Criteria to guide ME/CFS diagnosis [12] (NB: the earlier pilot study used the Canadian Criteria, which was replaced by the International Criteria in 2011–12). To be included in the ME/CFS participant cohort, the International Consensus Criteria must have been satisfied.

All participants performed a test for orthostatic intolerance (standing test—see section on weighted standing time for details) that included the collection of autonomic data during repose and the standing task [13]. After the standing test, non-fasting venous blood samples were collected for routine pathology testing, in addition to a parathyroid hormone (PTH), thyroid function testing (TFT), vitamin D and serum activin B [8]. For participants who were able, 24-h urine samples were collected and the volume, sodium (Na^+^), potassium (K^+^) and creatinine 24-h excretion rates were calculated.

Qualitative symptom inventories and questionnaires were also conducted for each participant, including the Epworth Sleep Scale [14] and the DASS-42 [15,16].

For the range of tests conducted, please refer to previous publications describing the CFS Discovery pilot studies [8,13].

### 2.3. Data Cleaning, Organisation and Structure

Data were collected for each participant as standard practice for the CFS Discovery staff and stored electronically in the secure clinic database. Each participant/patient file contained all the questionnaire and survey data, the printed pathology results (Australian Clinical Laboratories, South Australia), the standing test (orthostatic intolerance) data, including blood pressure (BP), heart rate (HR) and associated autonomic measurements and calculations, the standing time and standing difficulty, as well as clinical notes recording patient details (age, sex, weight, height).

An identification code was assigned to each research participant by CFS Discovery staff, after which data were matched and added to a spreadsheet for researcher interrogation. Heart rate (HR) data collected during the standing test was assessed for evidence of POTS (postural orthostatic tachycardia syndrome), with a HR increase of ≥30 beats per minute (bpm) upon standing from a lying position accepted as positive for co-morbid POTS [13,17]. The final data collection included the standing time and difficulty scores, WST calculations, POTS (yes or no), blood, serum and urine pathology results, serum activin B, DASS and Epworth Sleep scale results, along with notes on other conditions or comorbidities.

After the clinic appointment, the participants were asked to collect a 24-h urine sample within a week of the clinic visit. A minority of participants did not collect this sample, resulting in a number of missing values for urinary Na^+^, K^+^, creatinine and their 24-h excretion rates. With small to medium samples sizes, the median for each WST class was calculated and used to fill the missing values for each specific class.

### 2.4. Orthostatic Intolerance (OI) Assessment

Standing difficulty is a subjective ordinal scale developed by CFS Discovery clinicians, which with standing time (maximum of 20 min, recorded at two-minute intervals with autonomic measurements, as well as at repose before and after standing) is used to calculate the weighted standing time (WST). The standing difficulty scale ranges from 0 (no difficulty standing during 20 min upright) to 10 (extreme difficulty to maintain an upright stance). If the participant was not able to stand for at least 10 min, they were given a standing difficult score of 14. The participants who could maintain an upright stance for longer than 10 min, but not stand for the entire 20 min, were scored at 12 for standing difficulty. The standing difficulty scale has not been validated on other patient/participant populations.

### 2.5. Weighted Standing Time (WST)

The standing test procedure to assess orthostatic intolerance and detect POTS has been published previously [13], with a British study finding similar rates of POTS in a cohort from northern England [17]. Furthermore, the WST and its capacity to stratify ME/CFS severity, along with identify useful patterns in diagnostic markers, was recently published [18].

In brief, the WST takes the standing time (0–20 min, recorded at 2-min intervals) and weights this time with the subjective standing difficulty score, as described by the following equation:

*Weighted standing time (WST)* = *Time standing (mins)* × (1 − (*Difficulty*/14)) (1)

The WST, therefore, provides a proxy for ME/CFS severity and a response variable with which to investigate the significance of the predictor (independent) variables and their interactions. The results presented herein were generated from the analyses of WST severity classes, as summarised in Table 1. With the majority of the study participants able to stand for the entire 20 min of the orthostatic intolerance (OI) test, standing time alone was not an effective response variable.

### 2.6. Statistics and Machine Learning

#### 2.6.1. Statistical Analyses

All descriptive statistics, test (inferential) statistics and regression/correlation analyses were performed using SPSS (Version 22—IBM SPSS software, Chicago, IL, USA).

Prior to conducting the appropriate statistical analyses, all raw data collected for investigation were subject to a one-sample Kolmogorov–Smirnov (K-S) test to assess whether they fulfilled a normal distribution, with K-S results of *p* ≤ 0.05 indicating that the specific marker distribution was significantly different from a normal curve. Based on the K-S results (Table 2), statistical significance between two groups was estimated by a Mann–Whitney U test, and three or more groups by Kruskal–Wallis non-parametric tests. Jonckheere–Terpstra non-parametric tests were also applied where the groups were clearly ordinal. Descriptive results were presented as the median and 25th–75th interquartile range (IQR).

Significance was set at *p* < 0.05 for the two group comparisons using the Mann–Whitney U test, and also for comparisons across more than two classes in the Kruskal–Wallis (KW) test.

#### 2.6.2. Machine Learning

R statistical programming version 3.5.1 was used to run the recursive partitioning algorithms random forest (R library randomForest) and decision trees (R library rpart) [19,20]. Algorithm tuning was performed via the R caret package [21].

Random forest analysis (RFA) was performed using the WST classes summarised in Table 1b. Due to class imbalance and the relatively small overall sample size, the healthy controls were combined with mild ME/CFS cases (Table 1a) to create an adjusted WST class 0, and therefore provide a larger class sample size for subsequent RFA. Running the original WST classes (Table 1a) as the response of interest resulted in very poor class prediction, and as such, an ineffective model, in spite of attempts to compensate with class balancing R script. Future studies will benefit from larger sample sizes, particularly for healthy control cases.

All the RFA results presented herein used the three-class (WST) model to detect predictors of absent or mild ME/CFS symptoms (0), compared to moderate (1) or severe (2) symptoms (Table 1b).

Severe cases were characterised by their inability to remain upright for the full twenty minutes of the standing test for orthostatic intolerance. Missing values in the raw data were filled by the median for each WST category, prior to RFA. Missing data was most pronounced for the 24-h urine markers, with 15–20% missingness found due to test non-compliance after the CFS Discovery initial appointment. The total case numbers for the ME/CFS and healthy cohorts are summarised in Table 2. Individual missing values were also found for serum urea and electrolytes, and MCH.

Via algorithm tuning (caret), all RFA had the following features:

*mtry* = 4 (4 predictor variables tried for splitting at each node); *ntree* = 5000 (5000 decision trees grown to determine predictor variable rankings). With the following features included—*replace = TRUE* (cases are replaced during algorithm bootstrapping), and *importance = TRUE* (as well as Gini Index ranking, scores based on permutation ranking).

As well as the primary RFA to detect and rank predictors of ME/CFS severity via WST, bagging and boosting ensembles for a variety of algorithms were tested in parallel using R statistical programming via the caret and caretEnsemble packages [21,22]. Bagging and boosting are resampling methods used by the algorithm of interest to increase prediction accuracy through reducing variation, or correcting errors during the analysis. The analyses presented allowed for the comparison of machine learning methods, and therefore the assessment of the best analytical strategy for the dataset of interest.

**Figure 1:**
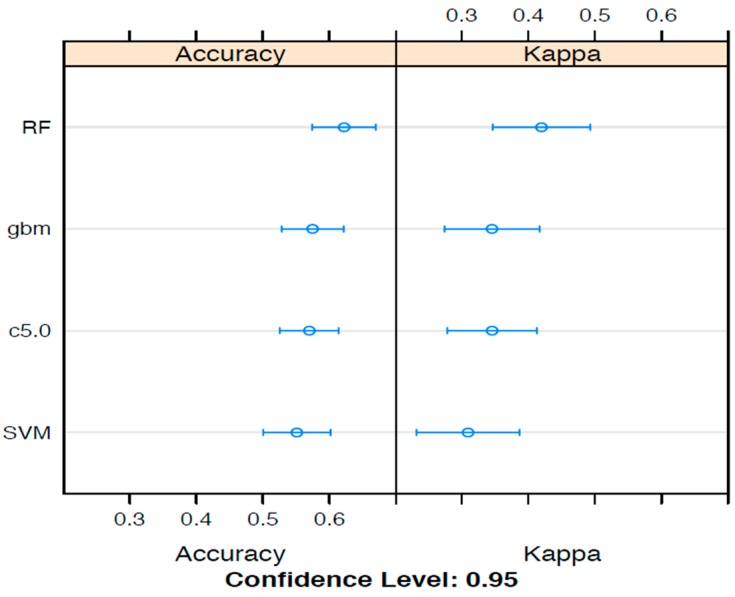
A variety of machine learning ensembles compared to assess performance for ME/CFS WST (0, 1, 2) class prediction, as measured by percentage accuracy and the Kappa statistic. Boosting strategies applied to enhance machine learning performance for ensemble random forest (RF), gradient boosting (gbm), c5.0 tree construction and support vector machines (SVM).

A number of machine learning options are available for the training and testing of data to reveal outcome predictors. To examine the best machine learning option, ensemble analyses that compared random forest analyses (RFA) to support vector machines (SVM), gradient boosting and decision trees, were conducted with the aims of assessing the comparative predictive accuracy of various machine learning techniques. The relationships between the various machine learning algorithm ensembles, presented as accuracy measures and kappa statistics, are summarised in Figure 1.

For the WST analyses, RFA produced the best accuracy and kappa results, suggesting this as the most suitable ML method to apply. For a comparatively small data set (for this study, 97 in total), RFA provides a method whereby hundreds to thousands of trees can be propagated as one analysis, and therefore introduce extra robustness into the analysis, which likely explains the superior performance for this data set. Nevertheless, the limitations of the total sample size did reflect in the large differences in accuracy and kappa statistic results. Receiver operating curves (ROC) and associated results were calculated by RFA modelling of MCH, ALP, serum urea, blood lymphocytes, 24-h urinary creatinine and activin B.

Random Forest Analyses (RFA) were subsequently applied to binary outcomes representing the direct comparison of ME/CFS to HCs, as well as the stratification of ME/CFS severity by WST (Table 1). Early investigations did not produce a model because of class imbalance between ME/CFS and HC categories, in spite of introducing class balancing script into the R code for RFA. Combining Healthy Controls with mild ME/CFS cases (Table 1b) solved this problem, allowing the building of RFA predictive models of disease categories. All the results presented hereafter are on the adjusted WST classes, as summarised in Table 1b.

To calculate the marker thresholds (e.g., ALP > or < 60 U/L), the recursive partitioning algorithm, decision trees, was used on the same dataset classified by WST, with trees developed also for the direct comparison ME/CFS to healthy controls, and the full WST classification from class 0–3 (Table 1a). For all the trees, the minimum split was 20 and the complexity parameter (*cp*) ranged from 0.01 to 0.085. The direct comparison of ME/CFS cases to healthy controls required a *cp* of 0.14. Due to the small to moderate starting sample sizes for each WST class, and that the final decision thresholds involved the loss of cases, results must be ascertained with caution, as the final decisions were often drawn from fewer than 10 cases.

### 2.7. Receiver Operating Characteristics

With the recognition of a predictor variable pattern by RFA, associated with the WST class, the diagnostic potential of the multi-marker profile to accurately separate ME/CFS severity was examined by receiver operating characteristic (ROC) curves, supported by an area under curve (AUC) calculation. A ROC curve plots assay sensitivity (rate of true positives) against the false positive rate (100—Specificity), with AUC estimating the accuracy of separating the two classes. As this suggests, only two WST classes were compared at one time, namely classes 0 versus 1, 0 versus 2, and class 1 versus class 2.

ROC plots were generated and AUC was calculated by the R statistical programming package ROCR [23].

Examples of R code and primary results generated by machine learning and ROC are available in the Supplementary Materials.

### 2.8. Activin B Assay

The development and optimisation of the activin B assay in human populations have been published previously [24,25]. However, for this study, the established assay for activin B was modified after it was discovered that non-specific interference was impacting the capacity of the assay to accurately measure lower activin B concentrations in human serum. The assay, which was used to measure serum activin B concentrations in the previous pilot study [8,18], was modified by the addition of activin-free gelding serum, as a carrier to remove the interference and enhance the accuracy of activin B detection.

## 3. Results

### 3.1. Direct Comparison of ME/CFS and Healthy Cohorts

**Figure 2:**
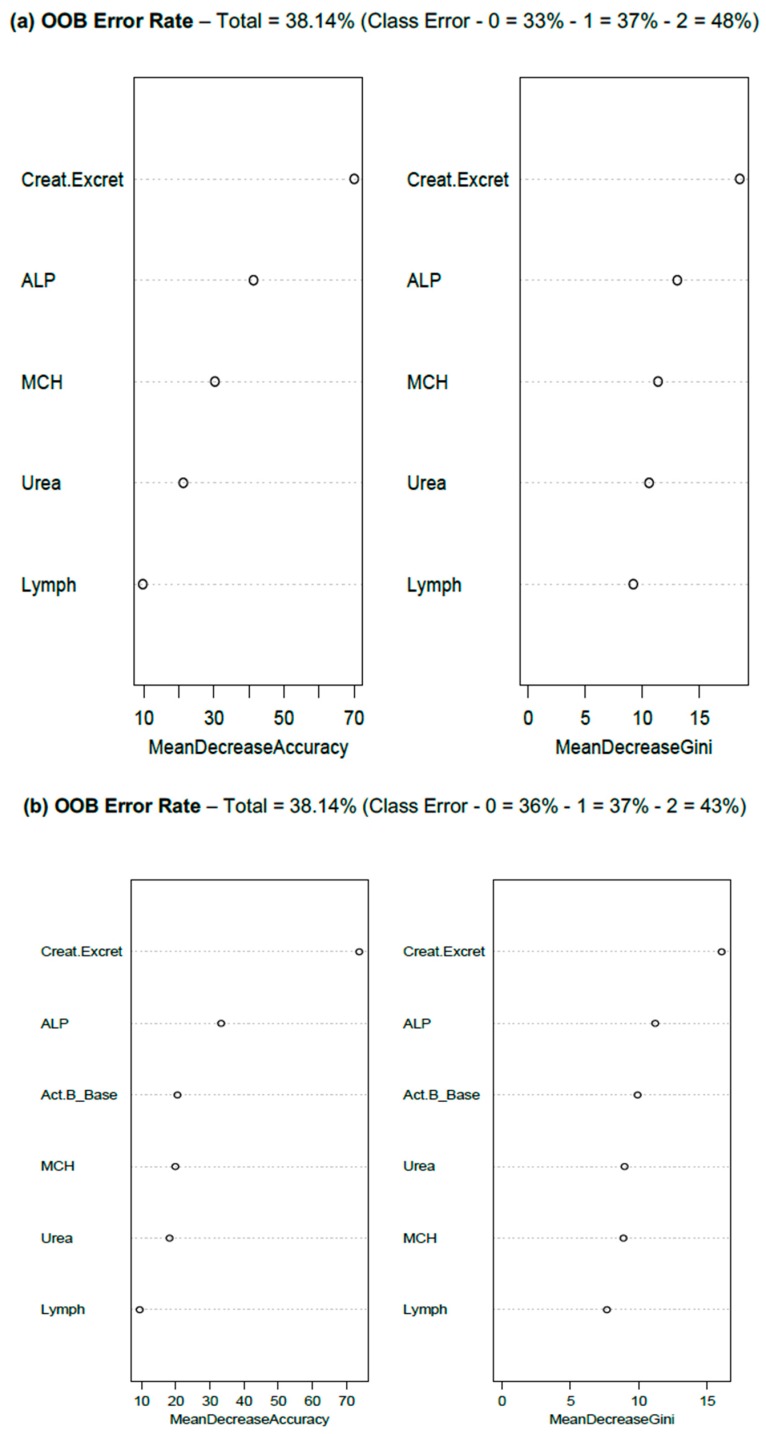
Random Forest plots of (**a**) a model using basic blood and urine test pathology markers to predict Weighted Standing Time (WST) classes (0, 1, 2) of ME/CFS, (**b**) the same model with Activin B results added. The left column represents the RFA Importance measure, and the right column the ranking of predictors by Gini Index. Refer to Table 1b for WST Class definitions. OOB—Out of Bag (Error Rate). Creat.Excret (24-h urinary creatinine excretion rate); ALP (serum alkaline phosphatase); Act.B_Base (first appointment, serum activin B assay); MCH (mean corpuscular haemoglobin); Urea (serum urea); Lymph (blood lymphocyte count).

The direct comparison of a range of pathology (blood, urine, serum) markers, questionnaire results and activin B are summarised in Table 2. The subset of pathology markers included were informed by exploratory data interrogation by machine learning (Figure 1 and Figure 2), with additional serum electrolytes, platelets, neutrophils and parathormone (parathyroid hormone—PTH) also included because of clinical interest in the potential importance of these markers, as well as for the association with renal function suggested by other results. Red cell indices and TFTs showed no anaemia or thyroid deficiency associated with chronic fatigue symptoms, and in general all individual pathology results from ME/CFS and HC were within the laboratory reference interval, with exceptions outside of the reference interval excluded from the analyses if clinically indicated as a diagnostic confounder.

As summarised in Table 2, the results of Kolmogorov–Smirnov (K-S) testing showed that platelets, ALP, neutrophils and age were assessed as being normally distributed, with the majority of markers at *p* ≤ 0.025, which therefore did not follow a normal distribution. For this reason, non-parametric statistics were used for all markers and survey results to determine whether statistical significance was achieved for comparisons between ME/CFS classes. The small loss of power due to nonparametric testing was regarded as clinically unimportant.

Statistically significant differences in median pathology results from the comparison of ME/CFS to HC cases were observed for serum urea, parathyroid hormone (PTH) and 24-h urinary creatinine excretion rate, with each of these significantly decreased for ME/CFS (*p* ≤ 0.05). The median age was significantly higher for the ME/CFS group, with the median total DASS score significantly elevated (separate depression and anxiety scores were significantly increased for the ME/CFS group, but not the stress score). Although sleep problems are often reported during ME/CFS assessment, the Epworth Sleep score did not differ significantly between the groups.

Activin B

An objective of this study was to validate a previous (pilot) study result, which found that activin B is a serum biomarker that significantly (*p* < 0.05) separates ME/CFS patients from healthy controls (HC). On direct comparison of the medians (Table 2), activin B was significantly lower (*p* = 0.013) for the ME/CFS cohort compared to results from the HC participant cohort. This is an inversion of the previous results, which found that activin B was significantly elevated in ME/CFS participants [8]. As described in the Materials and Methods, the activin B assay had been re-optimised prior to these analyses.

### 3.2. Analyses of Markers Stratified by Weighted Standing Time (WST)

#### 3.2.1. Four Severity Categories

Marker variation and survey results were investigated after the ME/CFS cohort was stratified by WST for symptom severity (classes 1–3) and compared to healthy controls (class 0) (Table 3). Median (25th–75th IQR—interquartile range) results were presented and statistical significance assessed by Kruskal–Wallis tests.

Serum urea, ALP and 24-h urinary creatinine excretion rate were statistically significant at *p* < 0.05. The difference between WST classes for DASS (Total) also achieved statistical significance, with increases in total DASS scores obvious for of the WST ME/CFS classes (1–3), when compared to HC (class 0).

Significance at *p* < 0.05 was not observed for activin B when comparing healthy controls (class 0) to the WST stratified ME/CFS cohort (classes 1–3) (Table 3). As seen in Table 3, apart from WST class 2 (moderate severity), the 25–75 IQR were large, suggesting high variations in the activin B results. When the healthy controls (WST 0) were compared directly to WST 2 by the Mann–Whitney U test, a significant result at *p* = 0.005 was found, whereas the comparison of WST 0 to WST 1 and 3 was not significantly different (*p* > 0.05). Based on this observation, activin B is most useful for separating healthy individuals from patients experiencing moderate ME/CFS symptoms, as defined by WST.

#### 3.2.2. Three Severity Categories

WST classes 0 and 1 were combined to increase sample size for subsequent machine learning (ML), resulting in adjusted WST classes representing categories defining absent or mild symptoms (0), moderate (1) or severe ME/CFS symptoms (2), as reflected by orthostatic intolerance. This adjusted WST classification (Table 1b) was used for all the following RFA and ROC investigations.

Age, Epworth Sleep Scale and total DASS score showed significant variations between WST classes (Table 4—Kruskal–Wallis test). Age was significantly higher for the ME/CFS cohort compared to healthy controls (Table 2). Comparison across WST classes indicated that the participants with moderate symptom severity were responsible for this age difference, which will require further investigation. Of the serum/blood markers, only MCH and ALP were significantly different, with ALP WST class 1 of a higher median compared to WST 0 and 3. Age can impact serum ALP levels; therefore, caution must be exercised when interpreting this result.

A significant difference between WST classes was not observed for activin B. The combination of healthy controls with mild cases increased the WST 0 median, and therefore statistically significant separation from WST classes 1 and 2 was not achieved.

### 3.3. Exploratory Machine Learning Analyses of ME/CFS and Healthy Control Data

As assessed by algorithm ensembles that calculated percentage accuracy and the kappa statistic (Figure 1), Random Forest Analysis (RFA) was chosen as the machine learning method to conduct deeper analyses of the ME/CFS results. Two sampling methods were tested for each ensemble, namely (a) boosting and (b) bagging. In general, similar accuracy and kappa results were found for both sampling strategies (bagging results not shown).

Figure 2 presents the results of two RFA, one with five routine pathology markers, and the other with activin B included in the same pathology model. The pathology markers represent the most effective constellation of blood or urine test results that most successfully predicted WST categories 0, 1 and 2, with an overall predictive accuracy of 62–65%. The addition of extra pathology variables either did not improve the accuracy of the model or reduced overall WST class predictive accuracy.

The addition of Activin B to the model did not change the overall accuracy of the RFA model, but did slightly improve the prediction accuracy for WST class 2 (severe), at the expense of a poorer WST class 0 prediction (Figure 2b). Activin B ranked as the third most important predictor of ME/CFS-WST categories, behind 24-h urinary creatinine excretion rate and ALP, both on the importance ranking and mean decrease Gini index (Figure 2).

RFA emphasised 24-h urinary creatinine clearance as a key predictor of WST classes, with ALP ranking as the second most important predictor from among the pathology markers. The subsequent analysis of the same data by a tuned (*cp* = 0.01, *minsplit* = 20) single decision tree confirmed the leading role of urinary creatinine as a ME/CFS predictor (decision tree code and results are available in the Supplementary Materials).

### 3.4. Receiver Operating Characteristic (ROC) Analyses and Discrimination of WST Categories by Activin B and Pathology Markers Post Random Forest

To assess the predictive value of the RFA models applying activin B, mean corpuscular haemoglobin (MCH), serum urea, lymphocytes, alkaline phosphatase (ALP) and urinary creatinine excretion rate to the prediction of ME/CFS, ROC curves were plotted and the area under curve (AUC) was calculated.

ROC curves and AUC calculations were examined as pairwise comparisons between WST classes (0-1, 0-2, 1-2). RFA and ROC were not reliable for the direct comparison of ME/CFS to healthy controls, due to data imbalance issues described elsewhere.

**Figure 3:**
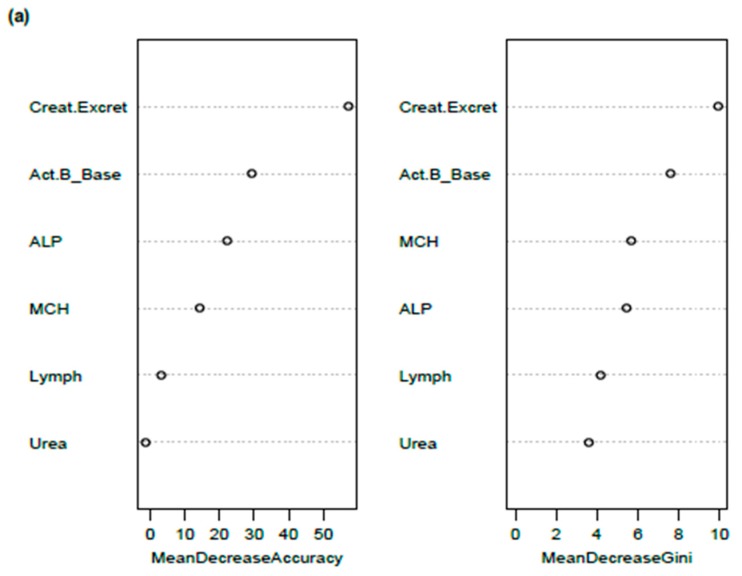

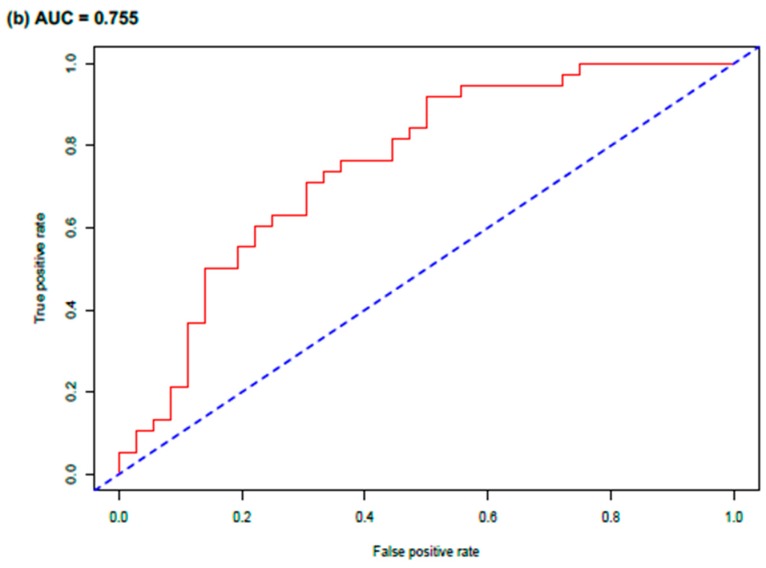
(**a**) Random Forest Analysis (RFA) and (**b**) Receiver Operating Characteristic (ROC) investigations on the prediction of Weighted Standing Time (WST) classes 0 versus 1, and the relative importance of activin B and pathology markers in separating the two WST classes of ME/CFS severity. AUC—area under curve (accuracy calculation from true and false positive rates).

Figure 3 presents the RFA and ROC results for the comparison of WST classes 0 and 1 (Table 1b). Figure 3a shows the Gini Index and Importance (Mean Decrease Accuracy) weighting of predictor variables to discriminate between WST classes 0 and 1 (mild symptoms and healthy cases combined versus moderate ME/CFS symptoms). The rate of urinary creatinine excretion was the top-ranked predictor, followed by serum activin B. For the total constellation of markers, the 0 versus 1 AUC was calculated at 0.755, with the ROC curve showing a clear separation from 0.50 (Figure 3b).

For the ROC-AUC analysis of class 0 versus class 2 (mild ME/CFS symptoms and healthy controls versus severe ME/CFS), urinary creatinine excretion rate was again the top-ranked predictor, with the impact of activin B reduced as determined by Gini Index and Importance scale, and serum urea and ALP elevated in predictive importance (AUC = 0.795). For classes 1 versus class 2, representing moderate versus severe ME/CFS symptoms, ALP, MCH, lymphocytes and serum urea ranked higher on both the Gini Index and Importance scale than urinary creatinine excretion and activin B, inverting the ranking observed for comparisons against class 0 (AUC = 0.704) (Results not shown).

### 3.5. Correct Prediction of ME/CFS Cases by RFA

**Figure 4:**
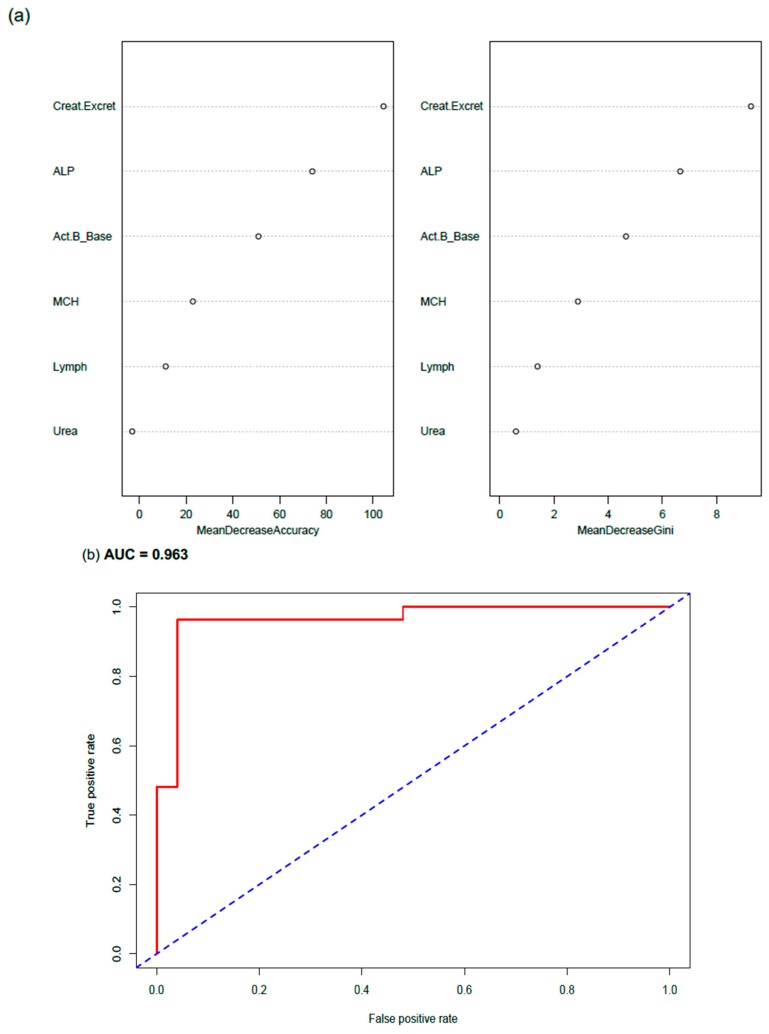
(**a**) Random Forest Analysis (RFA) and (**b**) Receiver Operating Characteristic (ROC) investigations on the prediction of Weighted Standing Time (WST) using only correctly predicted cases from the WST classes 0 versus 1, and the relative importance of activin B and pathology markers in separating the two WST classes of ME/CFS severity. Correct case prediction was performed via RF. AUC—area under curve (accuracy calculation from true and false positive rates).

As well as ranking predictors, the RF algorithm allowed the prediction of case category (WST class) based on the variables entered into the model. To understand the power of correctly predicted cases as a data modelling method to refine decisions on the diagnostic acuity of marker patterns, ROC was repeated for WST classes 0 versus 1, with only correctly RFA predicted 0 or 1 cases included (Figure 4). The importance ranking of predictors (Figure 4a) resembled that found for the all data general model (Figure 3), with urinary creatinine excretion rate, activin B and ALP the top three predictors of WST classes 0 or 1. The ROC curve showed an excellent separation from the 0.50 threshold, with an AUC of 0.963, which was clearly superior to AUC 0.755 found for the general model of the same WST classes that included all cases, regardless of correct prediction (Figure 3).

**Figure 5:**
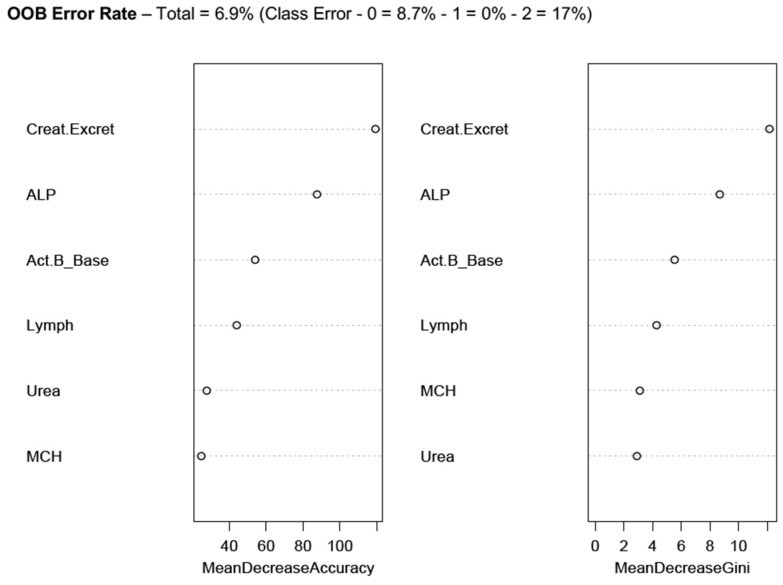
Relative predictor variable importance as determined by Random Forest Analysis (RFA) using only cases that were correctly predicted for each specific WST class (Table 1b) of ME/CFS severity. Correct case prediction by WST class was achieved by RFA.

The correctly predicted cases across the entire WST scale (Table 1b) were investigated by RFA to elucidate the broad pattern of the designated markers associated with the best accuracy prediction (Figure 5).

Similar to the ranking of markers for WST classes 0 versus 1 (Figure 3 and Figure 4), the urinary creatinine excretion rate, ALP and activin B were the top-ranked predictors of all the WST classes (Figure 5), which stratifies ME/CFS severity as according to orthostatic intolerance testing performance. While the cases were correctly predicted, WST class 0 recorded an (OOB) error rate of 8.7%, while class 2 recorded a 17% error rate. However, class 1 (Moderate severity) was perfectly predicted (Figure 5), suggesting again that the marker set including activin B is best for predicting symptom severity ranging from healthy, through mild, to moderate ME/CFS. The extent of the error rate in the severe cases indicates wider variation in these ME/CFS cases. Future studies involving larger participant samples will assist in determining predictive parameters with greater accuracy.

### 3.6. New Reference Intervals for Serum and Urine Markers Based on Correct Random Forest Prediction

To provide simpler and accurate guidance to clinicians supporting ME/CFS patients, reference intervals were calculated based on cases correctly predicted by RFA. The reference intervals were calculated using the median and 25–75% IQRs.

New reference intervals based on correctly predicted cases for each analyte of interest were calculated based on the following criteria: (1) comparison of the ME/CFS cohort with the healthy control group; (2) calculation of reference intervals following the WST criteria of categories 0 (healthy controls plus mild ME/CFS), 1 (moderate symptoms), and 2 (severe symptoms). The full WST classification definitions are summarised in Table 1.

Table 5 and Table 6 show the medians and 25–75th IQR for the ME/CFS predictors correctly detected by RFA (namely, MCH, lymphocyte count, serum urea, ALP, 24-h urinary creatinine excretion rate and activin B), using criteria 1 (Table 5) and 2 (Table 6).

The small sample sizes led to most markers having a significant overlap of ranges, and therefore not producing distinctive reference intervals. There were some exceptions, namely, for ME/CFS urinary creatinine excretion rate, a 25–75 IQR of 8.03–10.88 mmol/24 h (median—10.5), and 12.12–15.3 mmol/24 h (median—12.7) for healthy controls (Table 5).

For the WST comparison (Table 6), 25–75 IQR overlap was not found between classes for activin B, with separation of confidence intervals observed for MCH and urinary creatine excretion rate (between class 0 and class 1, as well as classes 0 and 2). There was a marginal separation of 25–75 IQR observed for serum urea classes 0 and 2. Using WST data for between class prediction and ROC analyses, the correctly predicted cases (by RFA) for classes 0 and 1 (Figure 4) emphasised the powerful role of 24-h urinary creatinine excretion rate as a potential diagnostic marker; it was the only marker analysed that showed a clear differentiation between class 0 in comparison to class 1 25–75 IQR intervals (results not shown).

The calculation of reference intervals specific to varying levels of ME/CFS severity, as quantitated by WST, was achieved (Table 5 and Table 6). With access to larger sample sizes, via large multi-centre studies and/or databases, the capacity to develop novel diagnostic guidelines using pathology results specific for ME/CFS, with and without activin B, will be possible.

Two multi-category, non-parametric statistical tests were used to assess significance for each predictor variable, the Kruskal Wallis (KW) and Jonckheere–Terpstra (J-T) Tests (Table 6). The methods are different in how they estimate significance across three or more classes, with the J-T test designed for investigations of ordered (ordinal) variables. While all variables were clearly significant (≤0.012) by the KW test, only serum urea, 24-h urinary creatinine excretion rate and activin B demonstrated significance for both tests, suggesting enhanced statistical robustness for these markers in terms of variation across the three WST classes.

## 4. Discussion

As demonstrated previously by us [8,18] and others [26], the results of pathology testing are not remarkable for ME/CFS patients, and often there are no statistically significant differences in the pathology results for ME/CFS when compared to healthy control subjects, although a recent study has identified the pathology test for creatine kinase (CK) as a significant marker to separate ME/CFS from control samples [27]. These difficulties in detecting quantitative markers for ME/CFS diagnosis have stimulated many investigations over the past 30 years, and with research consistently suggesting immune system involvement or dysfunction [28], cytokine studies have featured prominently in these attempts at biomarker development [1,2,3,4,5,6,7]. The search for a cytokine biomarker has been fraught with frustration, for example, the promise of a TGF-β marker was stymied by the realisation that sample preparation may explain serum concentration variation [7]. To this literature on putative ME/CFS serum biomarkers, we added activin B, which is useful in isolation, but also as a ratio with activin A or follistatin [8] and maintains statistical significance across WST classes [18].

The research presented here is a validation study on the potential of activin B as a reliable serum marker for ME/CFS, which would be a major advance in this field in light of the history of biomarker development. As reported, serum activin B showed statistical significance in separating ME/CFS participants from healthy controls, and additionally, demonstrated a capacity to differentiate WST classes in combination with select pathology markers. However, for this new research population, the trend was reversed, with healthy control participants showing a significantly increased median compared to the ME/CFS cohort. A cohort of existing CFS Discovery patients were recruited as research participants for this study, and issues associated with small to medium samples sizes may have contributed to these findings. The re-calibration of the activin B assay, due to sensitivity variation across the range of detection, improved the accuracy of the assay at lower serum concentrations, thus enhancing activin B detection capacity and broadening the reference interval range, which may explain the differences in activin B results found for this study when compared to the previous results [8].

Exploratory random forest analysis (RFA) was performed on the same data, with subsequent analyses focussed on WST data only (see Table 1b). The standard RFA of WST data (5000 trees per analysis, four predictor variables tested per node) resulted in (OOB) error rates of 38.14%. Activin B was also investigated as a member of a six-marker profile that included 24-h urinary creatinine excretion rate, mean corpuscular haemoglobin (MCH), alkaline phosphatase (ALP), serum urea, and total lymphocyte count. RFA, with or without activin B, showed an identical overall prediction error rate (OOB), but with the addition of activin B to the marker profile, a reduction in WST 2 (severe) class prediction error rate was identified, at the expense of an error rate increase for WST 0 (WST 1 error rate remained stable). For this RFA, urinary creatinine, ALP and activin B were the top predictors of WST class. The capacity of activin B to enhance discrimination between WST classes was also a feature identified by RFA.

Single decision trees confirmed the primacy of 24-h urinary creatinine clearance as a ME/CFS predictor, with a calculated decision threshold of 11.96 mmol/L separating class 0 (accuracy 83.3%) from classes 1 and 2, while ALP separated classes 1 and 2 at 62.5 U/L (accuracies of 86.4% and 75% respectively). Caution must be exercised when interpreting these results, since the final accuracy scores were often calculated from ≤20 cases.

As an extension of RFA, the panel of six predictive markers was assessed by receiver operating characteristic (ROC) curves to investigate the impact of test profile sensitivity and specificity (false negative, false positive rates). Pairwise WST classes were analysed per ROC, both for the entire data set, and for the correctly predicted cases for each WST class (0, 1, 2). Activin B remained in the top three in terms of predictor importance, with the model producing an AUC of 0.76 for all cases and an AUC of 0.963 for models comprising only correctly predicted outcomes. The correctly predicted cases from each WST class were subsequently used to calculate new reference intervals for each of the six RFA predictors (Figure 2).

Due to the broad reference intervals calculated as medians and 25th–75th IQRs, distinct separation between WST classes was not common, but did feature for 24-h urinary creatinine clearance and some class comparisons for activin B. As stated earlier, larger participant cohorts are required for validation, and the calculation of accurate reference intervals via the method presented here.

In tandem with research on ME/CFS immunology, pathology and cytokine biology, metabolomics is yielding valuable insights into ME/CFS aetiology [29,30], which in turn crosses into mitochondrial function [31]. New and sophisticated evidence of mitochondrial dysfunction in ME/CFS patients has emerged recently from patients involved as research participants in this project, with blast lymphocytes grown from blood samples collected at CFS Discovery and analysed via *Seahorse* technology [32].

Potential exists to meld metabolomics with immunity, and mTOR (mammalian targets of rapamycin and TORC subunits), which has a role in amino acid transport and protein synthesis [33], may be central to this link, particularly in the context of muscle growth [34,35]. Muscle pain and weakness are often reported as leading ME/CFS symptoms [12,18]. TGF-β and activin proteins involve mTOR interaction, including for natural killer (NK) cells [36], which are regularly noted as deficient in ME/CFS patients [37], as well as for cartilage and bone biology [38]. Separating the specific biology of activin B from the well-studied roles of activin A has shown insights in relation to SMAD signalling [10,39,40], which will further illuminate activin B utility in the context of ME/CFS.

The centrality of NK cells to ME/CFS has been challenged recently by a comprehensive study involving more than 300 total participants, which included healthy and fatigue controls, as well as participants with varying levels of ME/CFS symptom severity [41]. NK cell numbers and function, as reflected by subtype proportions or responsiveness post in vitro stimulation, were not different between the control and ME/CFS cohorts. Instead, CD8^+^ T-cell proportions were altered, and mucosal associated invariant T cells (MAIT) increased for ME/CFS.

Future investigations will present results from the interrogation of databases that contain activin, pathology, mitochondrial and metabolomics results, and thereafter assist in the identification of additional immune-metabolomic biomarker patterns. Such results will thereafter contribute to the elucidation of disease mechanism via the unravelling of impaired metabolomic pathways, and understanding of the subsequent impact on immune function, muscle physiology and neurophysiology for ME/CFS patients.

In conclusion, activin B retained the capacity to separate ME/CFS cases from healthy controls (Table 2), but as an inverse relationship compared to the situation reported previously [8], with healthy controls having a higher median. The potential, therefore, to develop activin B as a general serum marker of ME/CFS needs multi-centre studies with large participant cohorts. While the current project recruited 97 participants, these were spread across the spectrum of good health to severe ME/CFS symptoms, hence resulting in small to moderate samples sizes. RFA studies revealed the unexpected role of activin B as a useful supporting marker for the discrimination of mild to moderate ME/CFS symptoms, as reflected by WST class, while severe cases were more difficult to predict via multi-marker RFA and the other methods developed to predict ME/CFS.

## Figures and Tables

**Table 1:** Definitions of ME/CFS symptom severity classes by Weighted Standing Time (WST), with reference to standing time and difficulty. (**a**) Four categories featuring a healthy control category, and a severity scale from mild to severe symptoms. (**b**) Three severity classes where the healthy control cohort (0) was combined with the mild category (1) from Table (**a**) to increase sample size for random forest algorithm interrogation (WST). All ME/CFS cases fulfilled the ICC diagnostic criteria.

**(a)**			
**Category**	***n***	**WST**	**Definition of ME/CFS Symptom Severity Class (Post ICC)**
0	17	14.29–18.57	Healthy—No disease; All stood 20 min at Difficulty 1–4
1	19	15.71–18.57	Mild severity; All stood 20 min at Difficulty 1–3
2	38	6.43–14.29	Moderate Severity; All stood 20 min at Difficulty 4–10
3	23	0.0–2.57	Severe; All <20 min standing + Difficulty 12 or 14
**(b)**			
**Category**	***n***	**WST**	**Definition of ME/CFS Symptom Severity Class (Post ICC)**
0 *	36	14.29–18.57	Healthy + Mild symptoms; All stood 20 min at Difficulty 1–4
1	38	6.43–14.29	Moderate Severity; All stood 20 min at Difficulty 4–10
2	23	0.0–2.57	Severe; All <20 min standing + Difficulty 12 or 14

ICC—International Consensus Criteria (Diagnostic criteria for ME/CFS [12]). Standing time scale—0 to 20 min, with measurements at every two minutes; Standing difficulty scale—0 represents no difficulty, 14 represents extreme difficulty resulting in the inability to stand upright for a minimum of 10 min. * (0 + 1 from Table 1a).

**Table 2:** Comparison of ME/CFS and healthy control participants via a range of pathology markers, questionnaire results, and serum Activin B. Results summarised as medians and 25th–75th IQR for a ME/CFS cohort diagnosed by the International Clinical Criteria, and a control cohort of healthy participants.

**Blood/Serum/Urine Marker**	**Median (25–75%)**		*p*-Value *	K-S (*p*-Value) **
	**ME/CFS (*n*)**	**Healthy Control (*n*)**		
MCH (pg)	30.80 (79) (29.40–31.50)	30.35 (17) (29.55–31.40)	0.44	0.025
Lymphocytes (×10^9^/L)	2.00 (80) (1.50–2.30)	2.30 (17) (1.90–2.60)	0.08	0.009
Neutrophils (×10^9^/L)	3.70 (80) (2.80–4.78)	4.30 (17) (3.35–5.35)	0.16	0.076
Platelets (×10^9^/L)	260.5 (80) (232.0–303.8)	264.0 (17) (234.5–297.0)	0.76	0.200
Serum Sodium (mmol/L)	141 (79) (140–142)	141 (17) (139.5–142)	0.15	<0.001
Serum Bicarbonate (mmol/L)	29 (79) (27–32)	29 (17) (28–30.5)	0.32	0.016
Serum Urea (mmol/L)	4.9 (79) (3.9–5.7)	5.5 (17) (4.8–7.9)	0.04	0.002
Serum Creatinine (µmol/L)	74.0 (79) (66.0–82.0)	76.0 (17) (67.5–82.5)	0.67	0.001
ALP (U/L)	67.5 (80) (56.0–80.5)	65.0 (17) (52.0–77.5)	0.49	0.200
PTH (pmol/L)	5.45 (80) (3.33–6.78)	7.70 (15) (5.20–8.80)	0.03	0.001
Urinary Creatinine Excretion Rate (mmol/24 h)	9.9 (68) (8.1–11.7)	13.1 (13) (10.8–17.2)	0.004	0.020
Activin B (pg/mL)	85.95 (80) (70.48–125.76)	114.19 (17) (92.21–162.24)	0.013	<0.001
**Survey Results**	**ME/CFS (*n*)**	**Healthy Control (*n*)**	*p*-Value *	K-S (*p*-Value) **
DASS (Total) ^	28.0 (54) (14.8–47.0)	8.0 (17) (4.0–11.5)	<0.001	0.001
Epworth Sleep Scale	5.5 (54) (3.0–9.3)	4.00 (17) (3.0–6.5)	0.09	<0.001
Age (Years)	48.0 (80) (39.3–56.0)	41.0 (17) (29.0–51.0)	0.02	0.200

* Median and 25–75% IQR (Inter-Quartile Range) - Mann–Whitney U test - Significance at *p* < 0.05. ** Kolmogorov–Smirnoff test (one sample) for Normal data distribution. ^ DASS-42 (Depression, Anxiety, Stress total score).

**Table 3:** Comparison of pathology markers, questionnaire results and serum Activin B for ME/CFS sub-cohorts stratified by the Weighted Standing Time (WST) symptom severity scale, compared with a control cohort of healthy participants. Results summarised as medians and 25th–75th IQR.

**Blood/Serum/Urine Marker**	**Weighted Standing Time Median (25th–75th)**				*p*-Value *
	**WST 0 (*n*)**	**WST 1 (*n*)**	**WST 2 (*n*)**	**WST 3 (*n*)**	
MCH (pg)	30.8 (17) (29.4–31.5)	30.6 (19) (30.0–31.2)	30.3 (38) (28.7–31.0)	30.9 (23) (30.2–31.6)	0.08
Lymphocytes (×10^9^/L)	2.3 (17) (1.9–2.6)	1.8 (19) (1.5–2.8)	2.0 (38) (1.5–2.3)	2.1 (23) (1.8–2.5)	0.10
Neutrophils (×10^9^/L)	4.3 (17) (3.4–5.4)	3.6 (19) (2.6–4.8)	4.1 (38) (2.9–4.9)	3.5 (23) (2.5–4.5)	0.24
Platelets (×10^9^/L)	264 (17) (235–297)	244 (19) (203–308)	258 (38) (227–307)	273 (23) (251–300)	0.50
Serum Sodium (mmol/L)	141 (17) (139.5–142)	140.8 (18) (141.5–142)	141 (38) (141–142)	140 (23) (141–142)	0.08
Serum Bicarbonate (mmol/L)	29 (17) (28–30.5)	30 (18) (27–32.3)	29 (38) (28–31.5)	29 (23) (27–30)	0.46
Serum Urea (mmol/L)	5.5 (17) (4.75–7.9)	5.1 (18) (3.8–5.6)	5.4 (38) (4.0–6.1)	4.5 (23) (4.0–5.1)	0.04
Serum Creatinine (µmol/L)	76 (17) (67.5–82.5)	73.5 (18) (68–93)	76 (38) (66.5–85.5)	71 (23) (65–77)	0.26
ALP (U/L)	65 (17) (52–77.5)	64 (19) (53–78)	72 (38) (62–84.5)	60 (23) (45–74)	0.035
PTH (pmol/L)	7.7 (15) (5.2–8.8)	5.7 (19) (3.7–6.8)	5.6 (38) (3.7–7.5)	5.2 (23) (2.9–6.5)	0.12
Urinary Creatinine Excretion Rate (mmol/24 h)	13.1 (13) (10.8–17.2)	10 (16) (7.8–14.4)	9.4 (31) (7.7–12.2)	10.2 (21) (9.2–10.9)	0.035
Activin B (pg/mL)	114.19 (17) (92.21–162.24)	89.48 (19) (59.97–147.17)	79.97 (38) (71.00–106.97)	89.74 (23) (70.48–133.19)	0.07
**Survey Results**	**WST 0 (*n*)**	**WST 1 (*n*)**	**WST 2 (*n*)**	**WST 3 (*n*)**	*p*-Value *
DASS (Total) ^	8 (17) (4–11.5)	25 (16) (10.3–36.3)	28 (26) (15.3–47.3)	28 (12) (16.3–54.5)	<0.001
Epworth Sleep Scale	4 (17) (3–6.5)	4 (16) (1.5–5.8)	7 (26) (3–12)	6.5 (12) (3.3–10.8)	0.04
Age (Years)	41 (17) (29–51)	45 (19) (39–50)	55 (38) (43–61.5)	42 (23) (36–53)	0.01

Weighted Standing Time (symptom severity scale during standing test)—WST 0 (Healthy Controls); WST 1 (ME/CFS - Mild); WST 2 (ME/CFS - Moderate); WST 3 (ME/CFS - Severe)—See Table 1a. IQR (Inter-Quartile Range). * *p*-value set at *p* < 0.05 (Bonferroni correction not required for Kruskal–Wallis tests). ^ DASS-42 (Depression, Anxiety, Stress total score).

**Table 4:** Comparison of pathology markers, questionnaire results and serum Activin B for ME/CFS sub-cohorts stratified by the Weighted Standing Time (WST) symptom severity scale, including a control cohort of healthy participants. WST 0 and 1 (Table 2) data were pooled prior to analysis. Results summarised as medians and 25th–75th IQR.

**Blood/Serum/Urine Marker**	**Weighted Standing Time Median [25th–75th]**			*p*-Value *
	**WST 0 (*n*)**	**WST 1 (*n*)**	**WST 2 (*n*)**	
MCH (pg)	30.8 (36) (29.9–31.4)	30.2 (38) (28.8–30.9)	30.9 (23) (30.2–31.6)	0.03
Lymphocytes (×10^9^/L)	2.0 (36) (1.6–2.6)	1.95 (38) (1.5–2.2)	2.1 (23) (1.8–2.5)	0.15
Neutrophils (×10^9^/L)	3.9 (36) (2.8–5.2)	4.1 (38) (2.9–4.9)	3.5 (23) (2.5–4.5)	0.34
Platelets (×10^9^/L)	254.5 (36) (214.3–305.0)	256.5 (38) (219.5–305.8)	273.0 (23) (251.0–300.0)	0.45
Serum Sodium (mmol/L)	141 (35) (140–142)	141.5 (38) (141–142)	141 (23) (140–142)	0.07
Serum Bicarbonate (mmol/L)	29 (35) (28–31)	29.5 (38) (28–31.3)	29 (23) (27–30)	0.52
Serum Urea (mmol/L)	5.1 (35) (4.6–6.4)	5.4 (38) (3.9–6.1)	4.5 (23) (4.0–5.1)	0.08
Serum Creatinine (µmol/L)	74 (35) (69–84)	76 (38) (66.8–84.8)	71 (23) (65–77)	0.13
ALP (U/L)	64 (36) (53.3–77.5)	72.5 (38) (62–85.3)	60 (23) (45–74)	0.014
PTH (pmol/L)	5.9 (34) (4.7–8.4)	5.6 (38) (3.7–8.4)	5.2 (23) (2.9–6.5)	0.19
Urinary Creatinine Excretion Rate (mmol/24 h)	12.7 (29) (8.3–15.3)	9.4 (31) (7.7–12)	10.2 (21) (9.2–10.9)	0.13
Activin B (pg/mL)	103.36 (36) (78.57–148.78)	80.73 (38) (71.27–107.81)	89.74 (23) (70.48–133.19)	0.15
**Survey Results**	**WST 0 (*n*)**	**WST 1 (*n*)**	**WST 2 (*n*)**	***p*** -Value *
DASS (Total) ^	11 (33) (6.5–29.5)	28 (26) (15.3–47.3)	28 (12) (16.3–54.5)	0.004
Epworth Sleep Scale	4 (33) (3–6)	7 (26) (3–12)	6.5 (12) (3.3–10.8)	0.015
Age (Years)	43 (36) (36.3–50)	54.5 (38) (43–61.3)	42.00 (23) (36–53)	0.007

Weighted Standing Time (symptom severity scale during standing test)—WST 0 (Healthy Controls + mild symptoms - WST 1, Table 3); WST 1 (ME/CFS - Moderate); WST 2 (ME/CFS - Severe)—See Table 1b. IQR (Inter-Quartile Range). * *p*-value set at *p* < 0.05 (Bonferroni correction not required for Kruskal–Wallis tests). ^ DASS-42 (Depression, Anxiety, Stress total score).

**Table 5:** Median and 25–75 IQR calculated from ME/CFS or healthy control cases correctly predicted by Random Forest Analysis (RFA). ME/CFS cases ranged from mild to severe symptoms, as determined by the International Consensus Criteria [12] and the standing test.

Blood/Serum/Urine Marker	Median (25–75%)		*p*-Value *
	ME/CFS (*n* = 42)	Healthy Control (*n* = 11)	
MCH (pg)	29.6 (28.5–30.38)	30.8 (29.75–31.45)	0.44
Lymphocytes (×10^9^/L)	2.0 (1.48–2.23)	1.9 (1.5–2.6)	0.50
Serum Urea (mmol/L)	5.2 (3.9–5.75)	5.4 (4.65–6.35)	0.02
ALP (U/L)	78.5 (66.75–85.25)	60.0 (50.5–74.0)	0.17
Urinary Creatinine Excretion Rate (mmol/24 h)	10.5 (8.03–10.88)	12.7 (12.12–15.3)	<0.001
Activin B (pg/mL)	82.0 (71.26–104.86)	119.58 (89.49–167.37)	0.002

* Statistical significance *p* < 0.05. IQR (Inter-Quartile Range).

**Table 6:** Median and 25–75 IQR calculated from a combined class of healthy control and mild ME/CFS cases (WST 0), moderate (WST 1) and severe (WST 2) symptoms correctly predicted by Random Forest Analysis (RFA). ME/CFS was diagnosed via the International Consensus Criteria [12], and WST class calculated from standing test results.

Blood/Serum/Urine Marker	Weighted Standing Time Median (25th–75th)			*p*-Value *
	WST 0 (*n* = 23)	WST 1 (*n* = 23)	WST 2 (*n* = 12)	
MCH (pg)	30.8 (29.8–31.5)	29.5 (28.6–30.3)	30.95 (30.2–32.25)	(a) 0.006 (b) 0.811
Lymphocytes (×10^9^/L)	1.9 (1.5–2.3)	1.9 (1.4–2.1)	2.5 (2.1–3.0)	(a) 0.004 (b) 0.094
Serum Urea (mmol/L)	5.4 (4.6–6.4)	5.4 (3.9–5.9)	4.2 (3.83–4.58)	(a) 0.012 (b) 0.010
ALP (U/L)	60.0 (52.0–74.0)	79.0 (67.0–86.0)	50.5 (34.75–72.5)	(a) <0.001 (b) 0.840
Urinary Creatinine Excretion Rate (mmol/24 h)	13.1 (12.12–15.6)	10.50 (7.5–11.4)	10.45 (9.7–11.23)	(a) <0.001 (b) 0.001
Activin B (pg/mL)	130.36 (97.03–171.45)	81.48 (71.53–106.47)	93.86 (76.46–132.01)	(a) 0.004 (b) 0.013

* *p*-value set at *p* < 0.05 (Bonferroni correction not required for Kruskal–Wallis tests). *p*-values (**a**) Kruskal Wallis test (**b**) Jonckheere–Terpstra Test. IQR (Inter-Quartile Range).

## Supplementary Materials

The following are available online at https://www.mdpi.com/2075-4418/9/3/79/s1.
