# Supplementary material for: Rethinking ME/CFS Diagnostic Reference Intervals via Machine Learning, and the Utility of Activin B for Defining Symptom Severity
Source: Diagnostics (Basel). 2019 Jul 19;9(3):79. doi: 10.3390/diagnostics9030079 (PMC6787626; doi:10.3390/diagnostics9030079)
Supplement: Supplementary file 1 [file diagnostics-09-00079-s001.pdf]

Appendices – "Rethinking Reference Intervals …" (Lidbury *et al.*)

Appendix 1 –

Random Forest Code and Raw Results:

(a) Without Activin B:

Call: randomForest(formula = as.factor(WST_Cat.New) ~ MCH + Lymph + Urea + ALP + Creat.Excret, data = Act.CFS_tuning, keep.forest = T, replace = T, importance = T, ntree = 5000, mtry = 4, na.action = na.omit)

Type of random forest: classification

Number of trees: 5000

No. of variables tried at each split: 4

OOB estimate of error rate: 38.14%

Confusion matrix:

WST Class	0	1	2	Class Error
0 (Healthy/Mild)	**24**	8	4	0.333
1 (Moderate)	8	**24**	6	0.368
2 (Severe)	3	8	**12**	0.478

(b) With Activin B included in the Model:

Call: randomForest(formula = as.factor(WST_Cat.New) ~ MCH + Lymph + Urea + ALP + Creat.Excret + Act.B_Base, data = Act.CFS_tuning, keep.forest = T, replace = T, importance = T, ntree = 5000, mtry = 4, na.action = na.omit)

Type of random forest: classification

Number of trees: 5000

No. of variables tried at each split: 4

OOB estimate of error rate: 38.14%

Confusion matrix:

WST Class	0	1	2	class.error
0	**23**	9	4	0.361
1	7	**24**	7	0.368
2	1	9	**13**	0.435

(c) Single Decision Trees – Raw results and Example Plots:

Results for Decision Trees (that include Activin B 1st Appointment Result):

("Minsplit" = 20 for all trees) – ***Plots: see below (1 = ME/CFS 0 = Healthy Control)***

CFS Y/N Model (optimised CP) -

> prune(fit.ActB.CFS, cp = 0.14)

n= 97

node),	split,	n,	loss,	yval,	(yprob)
1)	root	97	17	1	(0.175, 0.825)
2) Creat.Excret	>=11.85	26	10	1	(0.385, 0.615)
4) Lymph	>=2.15	9	2	0	(0.778, 0.222) *
5) Lymph	< 2.15	17	3	1	(0.177, 0.824) *
3) Creat.Excret	< 11.85	71	7	1	(0.099, 0.901) *

(* denotes terminal node)

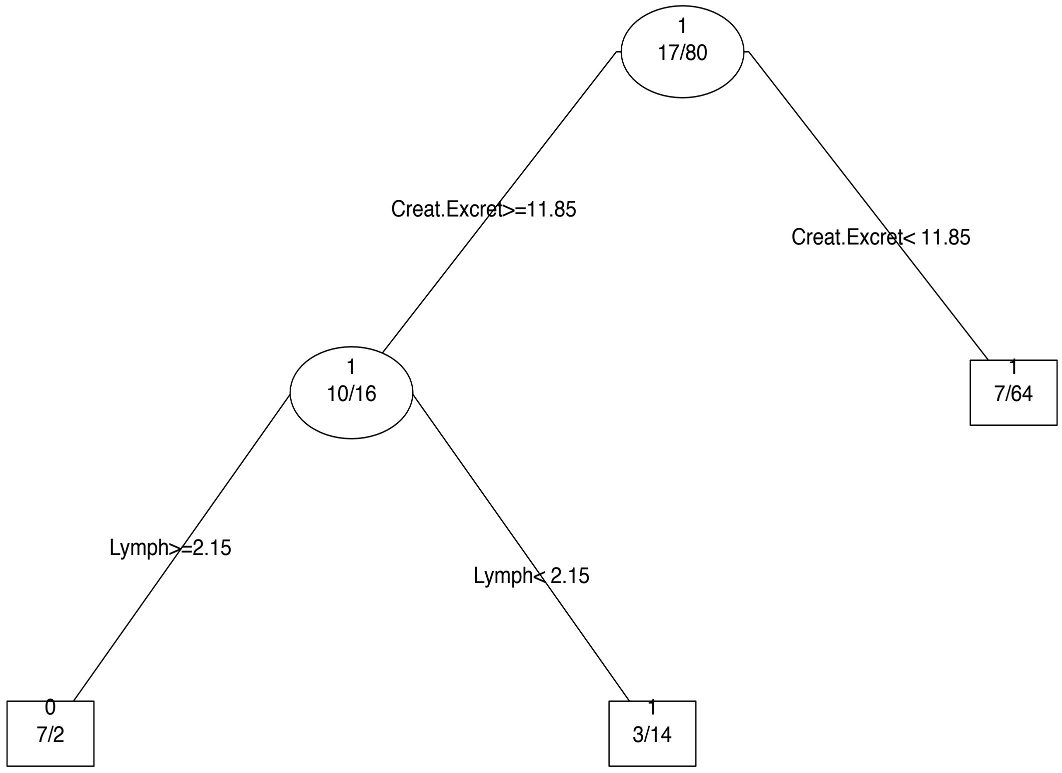
1
17/80
Creat.Excret>=11.85
Creat.Excret< 11.85
1
10/16
1
7/64
Lymph>=2.15
Lymph< 2.15
0
7/2
1
3/14

WST (0,1,2) (Cats 0 = Healthy/Mild, 1 = Moderate, 2 = Severe) Model (optimised CP) -

> prune (fit.ActB.CFS.WST3, cp = 0.01)

n= 97

node),	split,	n,	loss,	yval,	(yprob)
1)	root	97	59	1	(0.371 0.392 0.237)
2) Creat.Excret	>=12.45	23	8	0	(0.652 0.348 0.000)
4) MCH	>=30.6	10	0	0	(1.000 0.000 0.000) *
5) MCH	< 30.6	13	5	1	(0.385 0.615 0.000) *
3) Creat.Excret	< 12.45	74	44	1	(0.284 0.405 0.311)
6) Urea	>=5.35	27	11	1	(0.296 0.593 0.111)
12) Urea	>=7.55	8	3	0	(0.625 0.375 0.000) *
13) Urea	< 7.55	19	6	1	(0.158 0.684 0.158) *
7) Urea	< 5.35	47	27	2	(0.277 0.298 0.426)
14) ALP	>=80.5	9	2	1	(0.111 0.778 0.111) *
15) ALP	< 80.5	38	19	2	(0.316 0.184 0.500)
30) Creat.Excret	< 8.55	9	2	0	(0.778 0.111 0.111) *
31) Creat.Excret	>=8.55	29	11	2	(0.172 0.207 0.621) *

(* denotes terminal node)

WST (0,1,2,3 – Cats 0 & 1 Separate) Model (optimised CP) -

(Cat 0 = Healthy, 1 = Mild, 2 = Moderate, 3 = Severe)

> prune(fit.ActB.CFS.WST4, cp = 0.08050847)

n= 97

node),	split,	n,	loss,	yval,	(yprob)
1)	root	97	59	2	(0.175 0.196 0.392 0.237)
2) Creat.Excret	>=12.45	23	14	0	(0.391 0.261 0.348 0.000)
4) Act.B_Base	>=80.209	15	6	0	(0.600 0.267 0.133 0.000) *
5) Act.B_Base	< 80.209	8	2	2	(0.000 0.250 0.750 0.000) *
3) Creat.Excret	< 12.45	74	44	2	(0.108 0.176 0.405 0.311)
6) Urea	>=5.35	27	11	2	(0.185 0.111 0.593 0.111) *
7) Urea	< 5.35	47	27	3	(0.064 0.213 0.298 0.426)
14) ALP	>=80.5	9	2	2	(0.000 0.111 0.778 0.111) *
15) ALP	< 80.5	38	19	3	(0.079 0.237 0.184 0.500)
30) Creat.Excret	< 8.55	9	3	1	(0.111 0.667 0.111 0.111) *
31) Creat.Excret	>=8.55	29	11	3	(0.069 0.103 0.207 0.621) *

(* denotes terminal node)

Correctly Predicted WST (0,1,2) (Cats 0,1,2) Model (optimised CP) -

cp = 0.02857143

> prune(WST.Cats012.ActB.Path.TrPred.tree, cp = 0.02857143)

n= 58

node),	split,	n,	loss,	yval,	(yprob)
1)	root	58	35	0	(0.397 0.397 0.207)
2) Creat.Excret	>=11.96	24	4	0	(0.833 0.167 0.000) *
3) Creat.Excret	< 11.96	34	15	1	(0.089 0.559 0.353)
6) ALP	>=62.5	22	3	1	(0.000 0.864 0.136) *
7) ALP	< 62.5	12	3	2	(0.250 0.000 0.750) *

(* denotes terminal node)

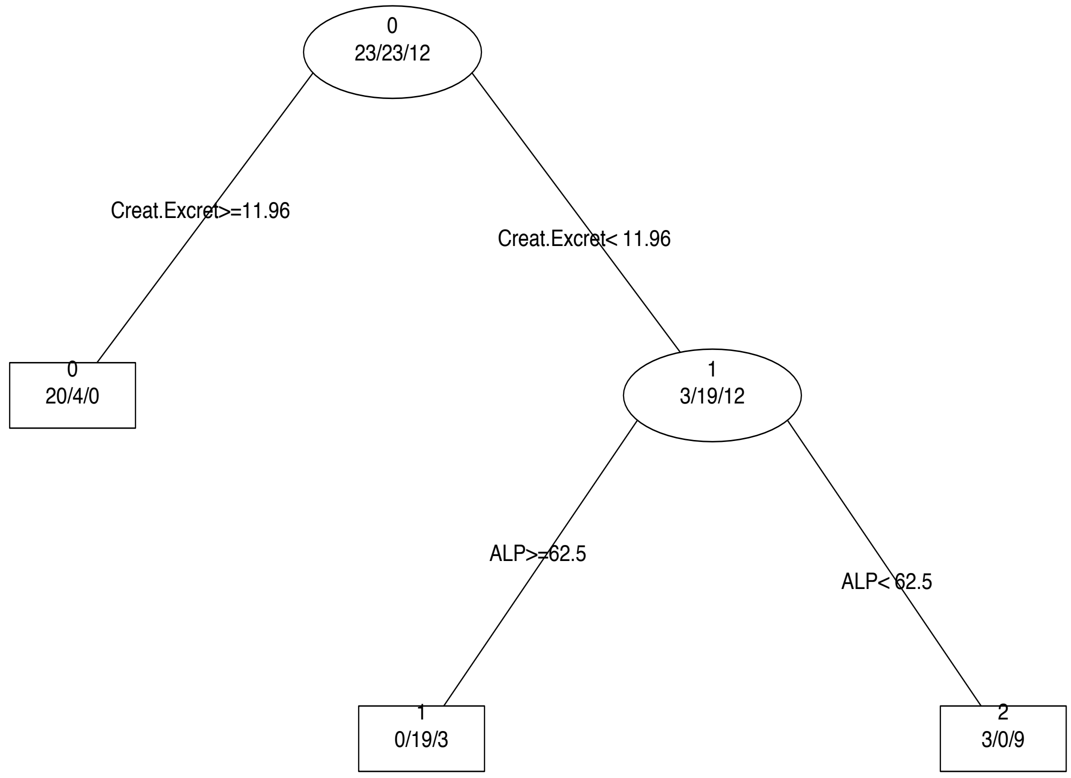

(d) R Code to support Receiver Operating Characteristic (ROC) curve and Area Under Curve (AUC) analyses:

ROC for WST (0,1,2) Categories 0 versus 1 (Healthy/Minor versus Moderate Symptoms)

```
> library (ROCR)
> Act.CFS_tuning_Cats.0.1.rf <- randomForest (as.factor(WST_Cat.New) ~ MCH + Lymph + Urea + ALP + Creat.Excret + Act.B_Base, data = Act.CFS_tuning_Cats.0.1, keep.forest = T, replace = T, method = "class", ntree = 5000, mtry = 4)
```

OOB estimate of error rate: 29.73%

Confusion matrix:

	0	1	class.error
0	25	11	0.3055556
1	11	27	0.2894737

```
> auc_ROCR <- performance (Act.CFS_tuning_Cats.0.1.rf.pred, measure = "auc")
> auc_ROCR <- auc_ROCR@y.values[[1]]
> print(auc_ROCR)
[1] 0.755117
```
